# Effects of Caffeinated Chewing Gum on Psychophysiological Responses and Kinematic Profiles During Intermittent and Continuous Small-Sided Soccer Games in Young Male Players: A Randomized Crossover Trial

**DOI:** 10.3390/nu18121962

**Published:** 2026-06-18

**Authors:** Bulent Kilit, Ersan Arslan, Yusuf Soylu

**Affiliations:** Faculty of Sport Sciences, Tokat Gaziosmanpasa University, Tokat 60250, Türkiye; ersan.arslan@gop.edu.tr (E.A.); yusuf.soylu@gop.edu.tr (Y.S.)

**Keywords:** caffeine supplementation, game-based training, exercise enjoyment, mental fatigue, locomotor responses

## Abstract

**Background/Objectives**: Caffeinated chewing gum is a practical, rapidly absorbed ergogenic aid increasingly used in team sports, yet its interaction with different small-sided soccer game (SSG) formats in young male players remains unclear. This study evaluated the effects of acute caffeinated (CAF) chewing gum on psychophysiological responses and kinematic profiles during intermittent (INT) and continuous (CON) 3-a-side SSGs. **Methods**: Twenty-four young male soccer players (18.4 ± 0.5 years) completed four 3-a-side SSG sessions separated by 48 h in a randomized, double-blind, placebo (PLA)-controlled, crossover design (CAF-INT, PLA-INT, CAF-CON, PLA-CON). Participants chewed 300 mg of CAF or PLA gum for 5 min, with mastication completed 5 min before warm-up session. The heart rates and kinematic profiles were recorded during the SSGs, and the ratings of perceived exertion (RPE), exercise enjoyment scale (EES), and visual analogue scale (VAS) to perceived mental fatigue (MF) were assessed post-game. **Results**: Compared with the PLA, the CAF increased the heart rate responses (HR), EES, total distance (TD), player load (PL), acceleration (ACC), and distances covered in selected speed zones (from Z0 to Z5), while reducing the RPE and MF. Significant format × supplementation interactions indicated that CAF-induced changes in high-intensity kinematic outcomes (TD, PL, ACC, Z2–Z5) and HR responses (HR_mean_, HR_max_) were generally greater in INT, whereas CAF-induced increases in low-intensity running distances (Z0 and Z1) and %HR_max_ were more pronounced in the CON format (all *p* < 0.05 for the reported effects; $\eta_{p}^{2}$ = 0.16–0.93 for CAF main effects [large effects]). The EES improvements were more pronounced in the CON format, whereas the MF and RPE reductions were more pronounced in the INT format. **Conclusions**: CAF chewing gum may be a practical acute strategy for modulating psychophysiological responses and kinematic profiles during SSGs, with the effects depending partly on the game format.

## 1. Introduction

Soccer is a physically demanding intermittent team sport requiring repeated high-intensity actions, sustained cardiovascular and neuromuscular loads, and continuous technical proficiency throughout match-play [1]. Successful performance also demands sustained decision-making capacity, as players must rapidly process and respond to constantly changing tactical situations [2]. Modern match analyses indicate that competitive players cover 10–13 km per game, with 8–12% performed at high intensity, and execute 150–250 brief explosive actions, including sprints, changes of direction, and jumps [3,4]. These repeated efforts, interspersed with incomplete recovery, impose substantial cumulative fatigue and demand on both physiological and cognitive resilience. Accordingly, strategies that help players maintain high-intensity performance and attenuate fatigue have become increasingly important in contemporary soccer preparation. Among the training modalities used to replicate these match demands, small-sided games have become particularly prominent.

Small-sided games (SSGs) are among the most frequently employed training modalities in contemporary soccer, simultaneously developing physical, technical, tactical, and psychological capacities within ecologically valid game-like scenarios [5]. These games are typically delivered in two structural formats: continuous (CON) games, involving uninterrupted play for a fixed duration, and intermittent (INT) games, comprising repeated bouts interspersed with rest intervals [6]. These formats elicit distinct psychophysiological and kinematic profiles. The INT formats typically generate greater high-speed running and sprint frequency because recovery intervals allow for partial restoration, whereas the CON formats may impose greater cumulative perceptual and cognitive demands [7,8]. Given these distinct demand profiles, the CON and INT formats may also respond differently to ergogenic interventions, particularly those with both central and peripheral mechanisms of action.

These distinct demand profiles may also provide a plausible mechanistic basis for format-dependent caffeine responsiveness. In INT-SSG, the presence of recovery intervals enables partial phosphocreatine resynthesis between bouts, creating repeated opportunities for caffeine’s peripheral mechanisms, including enhanced calcium release and reduced motor unit recruitment thresholds, to amplify high-intensity neuromuscular output [9,10]. By contrast, the uninterrupted nature of CON-SSG generates progressive adenosine accumulation and sustained cognitive load, potentially creating greater scope for caffeine’s central mechanisms to attenuate perceived effort and mental fatigue [9,10,11]. These considerations suggest that caffeine’s ergogenic profile may be differentially expressed depending on the SSG format [8,12]. Therefore, SSGs are widely used to manipulate internal and external training loads, determining whether acute nutritional strategies can modify responses to different SSG formats is of clear applied relevance.

To support such high training and match demands, evidence-based nutritional strategies have become integral to elite soccer preparation, as appropriately timed ergogenic aids can enhance performance, attenuate fatigue, and optimize recovery [13,14]. Caffeine is one of the most widely adopted and scientifically supported interventions in soccer, administered in approximately 97% of English professional clubs [15] and consumed by more than half of Premier League players on match days [16]. Its ergogenic effects are primarily through central adenosine A1 and A2A receptor antagonism, which attenuates fatigue signaling and enhances arousal, with additional peripheral actions on calcium release and neuromuscular excitability [9,10,17]. Among the available delivery formats, caffeinated (CAF) chewing gum has recently gained attention as a practical and rapidly bioavailable option. Approximately 80% of the dose is released within 5–10 min of mastication and absorbed through the buccal mucosa [9,12], enabling performance benefits as early as 15 min post-ingestion, which is considerably faster than the 45–60 min typically required for capsules, while minimizing gastrointestinal discomfort during high-intensity exercise [11]. These properties may make CAF chewing gum particularly well suited to the repeated high-intensity and cognitively demanding nature of soccer-specific training and match-play [18]. Furthermore, recent evidence from team sports, such as basketball and volleyball, suggests that CAF chewing gum may improve selected physical and sport-specific skill outcomes [19,20].

Soccer-specific investigations have examined CAF chewing gum across different age groups. In adult players, Ranchordas et al. [21] reported improvements in Yo-Yo Intermittent Recovery Test Level 1 performance and countermovement jump height following 200 mg ingestion, while Yildirim et al. [22] observed a 9% increase in maximum quadriceps strength following 200 mg of CAF chewing gum in caffeine-habituated male soccer players, whereas 100 mg had no significant effect, indicating that the ergogenic effects of CAF chewing gum may persist even in athletes with regular caffeine intake. In a younger cohort, Ellis et al. [23] showed that low doses of CAF (1–3 mg·kg^−1^, administered via capsule) enhanced the change-of-direction, sprint, and jump performance in elite young male players. More recently, Field et al. [24] found that acute ingestion of 200 mg of CAF chewing gum during simulated extra-time match-play attenuated reaction-time declines but reduced composure under pressure, highlighting the context-dependent nature of caffeine’s effects. Furthermore, Liu et al. [19] showed that 3 mg·kg^−1^ of CAF chewing gum improved the sprint performance, lower-limb power, shooting accuracy, and repeated-sprint fatigue resistance in basketball players, while Kaszuba et al. [20] reported that ~3 mg·kg^−1^ of CAF chewing gum improved the attack accuracy in volleyball players, although the physical performance variables did not change significantly. Despite this growing evidence, the interaction between CAF chewing gum and the SSG format remains unexamined in young soccer players. Prior SSG-based studies [25,26] have assessed caffeine’s effects on locomotor and cognitive performance, but none have manipulated the game structure. Of direct relevance, Soylu et al. [8] compared carbohydrate mouth rinse between INT and CON 4-a-side SSGs in adolescent players and reported a format-dependent signature: the CON format magnified reductions in perceived and mental effort, while the INT format amplified the enjoyment-enhancing effect. These findings suggest that the SSG structure may moderate the ergogenic response to acute nutritional interventions; however, it remains unknown whether a similar pattern exists for CAF chewing gum. Clarifying this interaction is important because CON and INT formats are routinely prescribed in applied settings, often without sufficient consideration of how their distinct demand profiles may interact with concurrent supplementation strategies.

Therefore, the aim of the present study was to evaluate the effects of acute CAF chewing gum supplementation on psychophysiological responses and kinematic profiles during INT and CON 3-a-side SSGs in young male soccer players. We hypothesized the following: (i) CAF chewing gum would enhance the HR responses and kinematic profiles and improve the perceptual–affective outcomes relative to a placebo (PLA) across both formats; and (ii) a significant format × supplementation interaction would emerge, whereby the psychological benefits would be greater in the CON format, while the kinematic benefits would be more pronounced in the INT format.

## 2. Materials and Methods

### 2.1. Participants

Twenty-four young male soccer players (age, 18.4 ± 0.5 years; stature, 176 ± 4 cm; body mass, 69.5 ± 4.2 kg; body mass index, 22.5 ± 0.6 kg/m^2^; Yo-Yo Intermittent Recovery Test Level 1 (YYIRT-1) distance, 1717 ± 414 m; estimated VO_2max_ from YYIRT-1, 50.8 ± 3.5 mL·kg^−1^·min^−1^; and training experience, 6.3 ± 1.2 years) voluntarily participated in this study. An a priori power analysis was conducted using G*Power (version 3.1.9.7; Heinrich Heine Universität Düsseldorf, Düsseldorf, Germany). Assuming a medium effect size (f = 0.25), an alpha level of 0.05, a statistical power of 0.80, and a correlation among repeated measurements of 0.5 for a within-participant repeated-measures ANOVA, the analysis indicated that a minimum sample of 24 participants was required [8]. The participants in this study were recruited by the investigators by screening team rosters according to inclusion and participation criteria and selecting potentially eligible individuals. The players were subsequently invited to an information session held at the club’s facilities, where the purpose of the study, the experimental procedures, the potential risks and benefits, and the voluntary nature of participation were explained in detail. Following the eligibility screening, 24 players met all inclusion criteria, agreed to participate, and provided written informed consent prior to enrolling in the study. Players were eligible if they (i) regularly engaged in structured soccer training at least four times per week, (ii) had a minimum of five years of competitive playing experience, and (iii) were familiar with SSG-based training. Players were excluded if they reported (i) any acute or chronic musculoskeletal injury or clinically relevant cardiovascular condition, (ii) the use of ergogenic substances or prescription medication during the previous month, or (iii) habitual daily caffeine intake exceeding 150 mg·day^−1^. Habitual caffeine consumption was quantified using a structured dietary questionnaire targeting the frequency of common caffeine-containing foods and beverages [27]. Before participation, all athletes received detailed information regarding the study procedures, potential risks, and expected benefits, and provided written informed consent. The study protocol was approved by the Institutional Ethics Committee of Tokat Gaziosmanpaşa University (Approval No.: 27.08.2025-614315; approval date: 27 August 2025) and was conducted in accordance with the Declaration of Helsinki.

### 2.2. Study Design and Procedures

**Figure 1:**
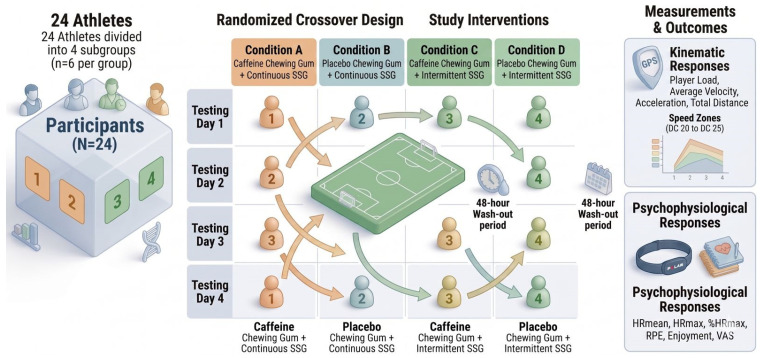
Schematic representation of the study design.

A randomized, double-blind, PLA-controlled, crossover study design was employed to investigate the independent and interactive effects of the game format (CON vs. INT) and CAF chewing gum supplementation (CAF vs. PLA) on psychophysiological responses and kinematic profiles during 3-a-side SSGs (Figure 1). The participants were allocated into four numerically balanced subgroups (*n* = 6 per subgroup) to counterbalance potential order effects, familiarization influences, and carry-over fatigue. Across four separate experimental days, each player completed, in a fully counterbalanced sequence, the following four conditions: CAF chewing gum combined with a CON-SSG (CAF-CON), PLA chewing gum combined with a CON-SSG (PLA-CON), CAF chewing gum combined with an INT-SSG (CAF-INT), and PLA chewing gum combined with an INT-SSG (PLA-INT). A 48-h wash-out interval separated every consecutive testing day to ensure adequate recovery and to minimize residual pharmacological effects. The allocation sequence was generated using a web-based randomization tool (www.randomizer.org) by an investigator who was not involved in the data collection or statistical analysis. To minimize potential learning effects and to ensure protocol familiarity, the participants attended a dedicated familiarization session prior to the experimental trials, during which they were introduced to the SSG formats, the chewing gum administration procedure, and the psychophysiological scales employed in this study.

All sessions were conducted at the same time of day to control for circadian variation in the physiological and perceptual responses [28]. The environmental conditions were monitored throughout all sessions and remained consistent across the trials (ambient temperature: 23 ± 2 °C; relative humidity: 50 ± 3%), minimizing any potential confounding effects of climatic variability [29]. Prior to enrollment, the athletes were confirmed as low habitual caffeine consumers (<150 mg/day) [27] and received a detailed list identifying common dietary sources of caffeine. For 48 h preceding each session, the participants were required to refrain from caffeine-containing foods, beverages, and supplements, and to avoid any strenuous physical exertion; alcohol consumption was likewise prohibited during the 24 h preceding each trial. To further reduce nutritional variability, each athlete recorded his food intake during the 24 h preceding the first trial and replicated it precisely before every subsequent session. A standardized 15-min warm-up session preceded each SSG session and was organized into three sequential 5-min blocks of low-intensity jogging, lower-limb dynamic stretching, and soccer-specific activation drills (progressive sprints, change-of-direction actions, and short passing sequences). Regarding the chewing gum intervention, players masticated either 300 mg of caffeine gum delivered through a commercially available CAF gum (Military Energy Gum, Ford Gum & Machine Company, New York, NY, USA) or a non-CAF-PLA gum (Orbit Peppermint/Spearmint, Wrigley Company, Mars, Türkiye) for a total of 5 min. The PLA gum was selected due to its similar pellet-shaped appearance, mint flavor, and chewing characteristics to the CAF gum. To assess blinding integrity, the participants were asked at the completion of the final trial to identify the supplementation condition received during each session. The proportion of correct guesses did not significantly exceed the chance levels, suggesting that blinding was successfully maintained throughout this study [20]. Gum ingestion was always scheduled to terminate 5 min before the onset of the warm-up [12]. To preserve blinding, all gums were distributed in unlabeled, pre-coded envelopes prepared by the supervising investigator, who was the only individual aware of the condition allocation; players were explicitly instructed not to disclose taste perceptions or subjective sensations, and the true nature of the supplementation conditions was revealed only after completion of the final trial. At the end of each experimental trial, athletes were briefly interviewed about any perceived side effects potentially associated with the supplementation, including gastrointestinal discomfort, palpitations, headache, or feelings of anxiety; no adverse events were reported at any point during the study period.

The SSG protocol was conducted on an artificial grass pitch measuring 20 × 30 m, yielding a constant relative playing area of 100 m^2^ per player. Two distinct game formats were compared: the CON format involved 18 min of uninterrupted play, whereas the INT format comprised six bouts of 3 min each, separated by 3 min of passive recovery. The selected SSG formats were based on previous studies comparing intermittent and continuous game structures for soccer players [6,7]. The total active playing time was matched at 18 min for both conditions to ensure that any differences between the formats reflected the presence or absence of recovery intervals rather than differences in exercise volume. Furthermore, bout duration has been identified as an important determinant of physiological and technical responses during SSGs [30]. Therefore, the INT format consisted of six 3-min bouts separated by 3-min passive recovery periods, whereas the CON format consisted of a single 18-min bout. To sustain a continuous flow of play and to elicit maximal efforts, multiple replacement balls were positioned around the pitch perimeter, and verbal encouragement provided by the coaching staff was standardized across all conditions [31]. All SSGs were played without goalkeepers, and each team used a small target goal (2 × 2 m) positioned at each end of the pitch. Within each 3-a-side team, players were assigned to positional roles—one forward, one midfielder, and one defender—using a randomized allocation procedure, and role assignments were maintained consistently across all four experimental conditions to minimize positional variability as a confounding factor [32].

### 2.3. Measurements

#### 2.3.1. Anthropometric and Aerobic Fitness Assessment

Anthropometric measurements, including stature and body mass, were obtained under fasting conditions prior to the first experimental session using a bioelectrical impedance analyzer (BC-418MA, Tanita Corp., Tokyo, Japan), which operates at a measurement frequency of 50 kHz. Aerobic capacity was subsequently evaluated via the YYIRT-1, following the acoustically paced progressive protocol described by Bangsbo et al. [33]. Throughout the test, heart rate (HR) was continuously tracked using a chest-worn monitor (Polar H10, Polar Electro, Kempele, Finland), and the peak HR value registered during the test was retained as the participant’s individual maximum. The estimated maximal oxygen uptake (VO_2max_) was then derived using the equation proposed by Bangsbo et al. [33]: VO_2max_ (mL·kg^−1^·min^−1^) = 36.4 + (0.0084 × YYIRT-1 distance in meters).

#### 2.3.2. Psychophysiological Responses

During all SSG sessions, HR was monitored continuously via a Polar H10 chest strap (Polar Electro OY, Kempele, Finland) operating at a transmission frequency of 2.4 GHz. Post-session data were processed to derive the mean HR and maximal HR, and the percentage of the maximal HR (%HR_max_) was calculated relative to each participant’s YYIRT-1-derived individual HR_max_. To minimize perceptual drift during high-intensity efforts, three subjective measures were obtained exactly 5 min after the cessation of each session. The rating of perceived exertion (RPE) was rated using the 15-point Borg RPE scale (ranging from 6 to 20) [34]; exercise-related enjoyment was quantified using the exercise enjoyment scale (EES), an 8-item instrument employing a 7-point Likert response format that has been validated among Turkish adolescent and adult athletes [35,36], and the subjective experience of mental fatigue (MF) was captured using a 100-mm horizontal visual analogue scale (VAS) anchored from 0 (“no MF”) to 10 (“extreme MF”) [37]. All questionnaires were completed individually in isolation from teammates to prevent any social contamination of the responses.

#### 2.3.3. Kinematic Profiles

External workload and time-motion characteristics were captured using 10-Hz Catapult OptimEye S5 Global Navigation Satellite System (GNSS) units (Catapult Innovations, Melbourne, Australia), each equipped with a 100-Hz triaxial accelerometer, a 100-Hz triaxial gyroscope, and a 10-Hz triaxial magnetometer. The validity and reliability of the device have been previously established [38]. The devices were worn on the upper back, secured between the scapulae by a custom-fitted vest, to ensure stable data acquisition during dynamic play. Following each session, the recorded data were exported and processed using the manufacturer’s proprietary software using the default filtering settings to compute the relevant kinematic indices, including the player load (PL, arbitrary units), total distance (TD, m), average velocity (AV, km·h^−1^), and the total number of accelerations (ACC, n). ACC were defined as events exceeding ±3.0 m·s^−2^. TD was further partitioned into six absolute velocity zones in accordance with prior work [39]: Z0 (0–11 km·h^−1^), Z1 (11.1–14 km·h^−1^), Z2 (14.1–17 km·h^−1^), Z3 (17.1–21 km·h^−1^), Z4 (21.1–24 km·h^−1^), and Z5 (>24.1 km·h^−1^).

### 2.4. Statistical Analyses

The normality assumption was assessed using the Shapiro–Wilk test. Descriptive data are presented as mean ± standard deviation (SD). To examine the main and interaction effects of the two experimental factors on the psychophysiological and kinematic profiles, a 2 × 2 repeated-measures analysis of variance (ANOVA) was conducted, with game format (CON vs. INT) and supplementation condition (CAF vs. PLA) as within-participant factors. Because both within-participant factors had only two levels, the sphericity assumption was inherently satisfied; therefore, neither Mauchly’s test nor the Greenhouse–Geisser corrections were required. When significant main or interaction effects were detected, the pattern of effects was characterized by an inspection of the estimated marginal means and the interaction contrast [(CAF-INT–PLA-INT)–(CAF-CON–PLA-CON)] reported with a 95% CI. The magnitude of the ANOVA effects were expressed as partial eta squared ($\eta_{p}^{2}$), with values of 0.01, 0.06, and 0.14 interpreted as small, medium, and large effects, respectively [40]. Statistical significance was set at *p* < 0.05. All statistical computations were performed in SPSS IBM SPSS Statistics version 27.0 (IBM Corp., Armonk, NY, USA).

## 3. Results

**Figure 2:**
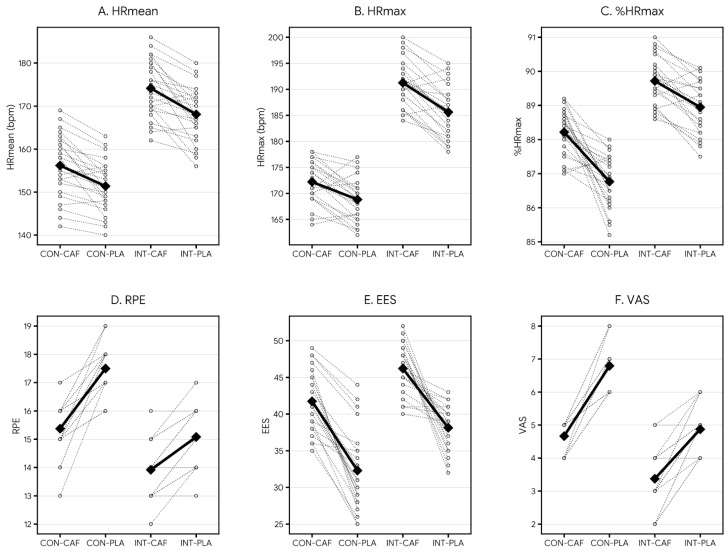
The CAF chewing gum intervention on psychophysiological responses during INT and CON 3-a-side SSGs: (**A**) HR_mean_; (**B**) HR_max_; (**C**) %HR_max_; (**D**) RPE; (**E**) EES; (**F**) VAS. Data are presented as mean ± SD.

The psychophysiological responses are presented in Figure 2. The INT format produced higher HR_mean_, HR_max_, %HR_max_, and EES than the CON format, whereas the RPE and VAS-rated MF were higher in the CON format. Compared with the PLA, the CAF chewing gum increased the HR_mean_, HR_max_, %HR_max_, and EES, and reduced the RPE and MF. Significant format × supplementation interactions were detected for the HR_mean_, HR_max_, %HR_max_, RPE, EES, and MF. The direction of these interactions differed across variables: CAF-induced increases in the HR_mean_ and HR_max_ were greater in the INT format, whereas the CAF-induced increase in %HR_max_ was greater in the CON format. The RPE and MF reductions were more pronounced in the INT format, whereas the EES improvements were more pronounced in the CON format. 

The kinematic profiles are summarized in Table 1. A significant main effect of the game format was observed for nearly all variables. The INT format yielded greater values than the CON format for PL, AV, ACC, TD, and distances covered from Z2 to Z5, whereas the CON format produced greater distances in Z0 and Z1. Compared with the PLA, the CAF chewing gum significantly increased PL, AV, ACC, TD, and distances covered at from Z0 to Z5. The CAF-induced increase in AV was likewise greater in the INT format than in the CON format, consistent with the significant format × supplementation interaction for this variable. The supplementation main effect for Z0 reflected an overall increase with CAF, with the magnitude of this increase being greater in the CON format than in the INT format. Similarly, the Z1 increase was markedly greater in the CON format than in the INT format, as reflected by the significant format × supplementation interactions for both zones.

Significant format × supplementation interactions emerged for PL, AV, ACC, TD, and distances at from Z0 to Z5 (Table 2). The pattern of these interactions was not uniform across the speed zones. For high-intensity outcomes (TD, PL, ACC, Z2–Z5), the magnitude of CAF-induced changes was generally larger in the INT format than in the CON format. By contrast, the CAF-induced increases in low-intensity running distances were markedly greater in the CON format: the Z0 increase was approximately five-fold larger in the CON format than in the INT format, and the Z1 increase was similarly greater in the CON format than in the INT format, both producing significant format × supplementation interactions (*p* ≤ 0.048). Notably, the %HR_max_ interaction also followed this pattern, with the CAF-induced increase being greater in the CON format (Table 2), in contrast to the HR_mean_ and HR_max_, for which the INT format showed larger CAF-induced changes.

## 4. Discussion

This study examined the effects of acute CAF chewing gum supplementation on psychophysiological responses and kinematic profiles during INT and CON 3-a-side SSGs in young male soccer players. The main findings were threefold: (i) the INT format elicited greater heart rate, EES, and high-intensity locomotor responses, whereas the CON format produced higher RPE and MF; (ii) CAF chewing gum improved HR responses, locomotor output, and EES, while reducing the RPE and MF; and (iii) a format-dependent pattern of caffeine responsiveness emerged, with high-intensity kinematic benefits (TD, PL, ACC, from Z2 to Z5) and HR responses (HR_mean_, HR_max_) generally greater in the INT format, whereas %HR_max_, low-intensity running distances (Z0 and Z1), and EES improvements showed greater CAF-induced increases in the CON format; MF and RPE reductions were more pronounced in the INT format. Together, these findings provide novel evidence that game format meaningfully shapes the ergogenic profile of CAF chewing gum in soccer-specific contexts.

### 4.1. Effects of Game Format on Psychophysiological and Kinematic Responses

The present findings demonstrate that the INT format induced greater HR strain and higher high-intensity locomotor output, as reflected by the increased PL, TD, ACC, and distances covered from Z2 to Z5. By contrast, the CON format produced higher RPE and MF, together with greater accumulation of low-intensity running (Z0 and Z1). These findings are consistent with the previous research indicating that INT-SSGs enable players to sustain higher work rates due to short recovery intervals that partially attenuate fatigue and facilitate repeated high-intensity efforts [7,8,13]. The elevated RPE and MF observed in the CON format likely reflect the cumulative perceptual and cognitive demands associated with uninterrupted exercise. Without recovery periods, players must continuously sustain attentional engagement, decision-making, and locomotor activity, which may increase subjective strain during sport-specific tasks requiring continuous cognitive and physical engagement [8]. Conversely, the higher EES observed in the INT format may be attributed to a more favorable balance between effort and recovery, allowing players to repeatedly perform high-intensity actions without prolonged perceptual burden. Taken together, these findings highlight that the SSG format selection meaningfully influences both the physiological load and subjective experience, and should therefore be aligned with specific training objectives.

### 4.2. Ergogenic Effects of Caffeinated Chewing Gum

The CAF chewing gum significantly enhanced the HR responses, locomotor output, and EES, while reducing the RPE and MF compared with the PLA. Notably, significant format × supplementation interactions for RPE and MF indicated that perceptual responses were influenced by both the game structure and the supplementation. The CON format elicited higher RPE and MF than the INT format, whereas the CAF reduced both variables compared with the PLA in both formats. These findings are consistent with a growing body of literature supporting caffeine as an effective ergogenic aid in soccer and team sport contexts [11,12,13,14]. The rapid onset of these effects is likely related to the pharmacokinetic characteristics of CAF chewing gum, as a substantial proportion of caffeine is absorbed via the buccal mucosa shortly after ingestion [12]. This rapid absorption may provide a practical advantage in situations where preparation time is limited, such as before warm-ups or during short breaks. The observed reductions in the RPE and MF are consistent with caffeine’s central mechanisms, including adenosine receptor antagonism and enhanced arousal [9,10,11]. Furthermore, the increase in the EES suggests that caffeine may positively influence affective responses, which are known to contribute to training adherence and motivation [41]. Collectively, these findings suggest that CAF chewing gum may be particularly useful during soccer-specific tasks that require repeated ACC, changes of direction, and intermittent high-intensity efforts.

Beyond soccer, similar ergogenic effects of CAF chewing gum have been reported in other team sports, such as basketball and volleyball. Liu et al. [19] showed that CAF chewing gum improved sprint performance, lower-limb power, shooting accuracy, and repeated-sprint fatigue resistance in basketball players. Kaszuba et al. [20] also reported improved attack accuracy in volleyball players, although the physical performance variables did not change significantly. These findings suggest that CAF chewing gum may support both physical and skill-related performance; however, its effects may vary according to the sport type, dose, timing, and task characteristics [19,20]. Importantly, the consistently large effect sizes observed across multiple variables ($\eta_{p}^{2}$ = 0.16–0.93) further support the practical relevance of these findings in applied soccer settings.

### 4.3. Interaction Between Game Format and Caffeine Supplementation

A key novel contribution of this study is the identification of a format dependent pattern in caffeine’s ergogenic effects, which was not uniform across the outcome domains. The HR_mean_ and HR_max_ were generally higher in the INT format than in the CON format, whereas the magnitude of the CAF-induced increase in %HR_max_ was greater in the CON format, and CAF elicited higher values than the PLA in both game formats. However, the magnitude of CAF-related changes differed across the HR variables. For perceptual–affective responses, CAF increased the EES and reduced the RPE and MF in both formats; the CAF-related improvement in the EES was more evident in the CON format, whereas the lowest absolute RPE and MF values were observed under the INT-CAF condition. One possible explanation is that the INT structure provides repeated opportunities for high-intensity output, allowing the caffeine-related enhancements in neuromuscular function and fatigue resistance to be more fully expressed. This interpretation is consistent with the previous findings demonstrating improved repeated-sprint and high-intensity performance following caffeine ingestion, with the effects reported across a range of habitual intake profiles [23,42]. While ergogenic responses appear particularly robust in low habitual consumers, a profile that matches the participants of the present study, statistically significant and practically relevant effects have also been documented in habituated athletes [24,43], suggesting that habitual caffeine intake may modulate, but does not necessarily abolish, the acute ergogenic response to CAF chewing gum.

By contrast, the greater psychological benefits observed in the CON format may reflect the higher uninterrupted perceptual load of this format, creating greater scope for caffeine to attenuate the perceived effort and fatigue under conditions characterized by sustained attentional demands and cumulative mental strain [8,44]. This is consistent with caffeine’s potential role in reducing the RPE and maintaining alertness during the CON type exercises [9,10,11]. A particularly notable finding was the format-dependent caffeine-induced increase in Z0 distance, which was more pronounced in the CON format than in the INT format. In the CON format, the absence of recovery intervals means that all locomotor activity, including low-intensity movement, occurs continuously throughout the game. Caffeine’s central stimulatory effects may therefore increase voluntary engagement and sustained movement even at lower intensities, amplifying Z0 accumulation particularly in the CON format. In the INT format, the preferential channeling of caffeine’s ergogenic effects toward higher intensity zones (from Z3 to Z5), combined with limited time for low-intensity locomotion within active bouts, may constrain Z0 accumulation relative to the CON format. These mechanisms are consistent with evidence that caffeine enhances overall activity levels across multiple intensity domains rather than merely shifting effort toward higher intensities [43]. However, as positional and time-resolved movement data (e.g., on-pitch vs. recovery-period activity) were not analyzed in the present study, these explanations remain speculative and should be interpreted with caution; future studies disaggregating active play from recovery intervals are warranted.

These findings extend the earlier work by Soylu et al. [8], who reported that the game format modulated the effects of carbohydrate mouth rinse on perceptual outcomes during SSGs. However, unlike mouth rinse, caffeine is believed to exert both central and peripheral effects, which may explain its greater impact on kinematic outputs in INT conditions. Nevertheless, the broader CAF chewing gum literature reveals task-dependent variability: while improvements have been reported in cycling [45], no significant effects have emerged in judo-specific performance [46]. These discrepancies indicate that the ergogenic effects of CAF chewing gum depend on the physiological, technical, and perceptual demands of the exercise task. In the present study, the effects of CAF chewing gum were more pronounced for the overall and higher-speed kinematic profiles (TD, PL, ACC, and from Z2 to Z5) in the INT games and for low-intensity locomotor accumulation (Z0 and Z1) in the CON games, while CAF increased the EES and reduced the RPE and MF in both formats.

### 4.4. Limitations

Several limitations should be acknowledged. First, the sample consisted exclusively of young male soccer players, limiting the generalizability of the findings to female athletes, younger populations, and elite-level players. Second, the relatively small sample size, although supported by an a priori power analysis, may limit the statistical power for detecting smaller interaction effects. Third, only low habitual caffeine consumers were included, which enhances internal consistency but restricts generalization to individuals with higher caffeine intake. Fourth, only a single caffeine dose and ingestion timing were examined, precluding a dose–response analysis. Finally, no physiological measures of caffeine concentration were obtained, limiting direct pharmacokinetic interpretation and precluding an analysis of individual differences in caffeine responsiveness, which the previous evidence suggests can be substantial even when group-level effects are limited [47]. Furthermore, the INT protocol employed in this study (6 × 3 min bouts with 3 min passive recovery) represents a specific and somewhat artificial exercise structure that may not fully replicate the demands of official soccer match play or typical training sessions, where the work-to-rest ratios are less clearly defined and recovery is rarely passive. Consequently, the generalizability of the present findings to competitive match conditions or ecologically representative training environments should be interpreted with caution. Future studies should address these limitations by exploring different populations, caffeine doses, timing strategies, habitual caffeine intake levels, and measurement approaches, as well as employing game formats more closely resembling official match structures to extend the external validity of these findings.

### 4.5. Practical Applications

From an applied perspective, CAF chewing gum (300 mg), administered shortly before warm-up, appears to be an effective and practical strategy for enhancing both physical performance and subjective exercise experience during SSG-based training. When the objective is to maximize high-intensity locomotor output and neuromuscular stimulus (TD, PL, and ACC, from Z2 to Z5), caffeine may be particularly beneficial when paired with INT-SSG formats. By contrast, when the training goal targets sustained low-intensity movement volume (Z0 and Z1) or HR load expressed as %HR_max_, the CON format may better potentiate caffeine’s effects. When the goal is to attenuate RPE and MF, caffeine offers greater perceptual benefits when paired with the INT formats, whereas the EES enhancements appear to be more pronounced in the CON formats. The chewing gum delivery format may also be useful in applied soccer settings because it is easy to consume, rapidly absorbed, and suitable for short preparation periods. Soccer coaches, strength and conditioning specialists, and sports nutritionists should nevertheless individualize caffeine use according to the athletes’ habitual caffeine intake, tolerance, and sensitivity to potential side effects.

## 5. Conclusions

The present study has demonstrated that both game format and acute CAF chewing gum supplementation meaningfully influenced the psychophysiological responses and kinematic profiles during 3-a-side SSGs in young male soccer players. The INT format elicited greater HR and high-intensity locomotor responses, whereas the CON format resulted in higher RPE and MF. The CAF chewing gum enhanced the HR and locomotor responses, increased the EES, and reduced the RPE and MF compared with the PLA. Crucially, the magnitude and domain of these ergogenic effects varied according to the game format: high-intensity kinematic benefits (TD, PL, ACC, from Z2 to Z5) and HR responses (HR_mean_ and HR_max_) were more pronounced in the INT format, whereas low-intensity running distances (Z0 and Z1) and %HR_max_ showed greater CAF-induced increases in the CON format; the EES improvements were more evident in the CON format, while the MF and RPE reductions were more pronounced in the INT format. Taken together, CAF chewing gum may be regarded as a context-dependent ergogenic strategy whose benefits are best realized when matched to the specific demands of the training task. From an applied perspective, these findings provide soccer coaches, strength and conditioning specialists, and sports nutritionists with a structured framework for aligning acute CAF chewing gum supplementation with SSG-based training objectives in young soccer players.

## Figures and Tables

**Table 1:** Kinematic profiles of the players during 3-a-side SSGs under different game formats and supplementation conditions (*n* = 24).

Variable	CAF-INT	PLA-INT	CAF-CON	PLA-CON
PL (A.U.)	229.25 ± 11.47	215.11 ± 8.39	193.98 ± 7.27	187.40 ± 6.77
AV (km·h^−1^)	7.20 ± 0.34	6.68 ± 0.34	5.13 ± 0.35	4.81 ± 0.33
ACC (*n*)	61.54 ± 6.90	56.33 ± 4.41	44.88 ± 4.79	41.25 ± 3.25
TD (m)	1763 ± 79	1635 ± 51	1588 ± 76	1508 ± 45
Z0 (m)	503 ± 36	496 ± 25	553 ± 28	516 ± 21
Z1 (m)	505 ± 22	501 ± 21	546 ± 20	510 ± 19
Z2 (m)	367 ± 23	345 ± 20	312 ± 17	298 ± 17
Z3 (m)	229 ± 25	205 ± 22	132 ± 9	126 ± 5
Z4 (m)	89 ± 11	71 ± 11	52 ± 10	42 ± 5
Z5 (m)	17 ± 3	11 ± 3	9 ± 3	5 ± 2

Note. Data are mean ± SD. CAF = caffeinated chewing gum; PLA = placebo chewing gum; CON = continuous small-sided game; INT = intermittent small-sided game; ACC = accelerations; PL = player Load; AV = average velocity; TD = total distance; Z = zone.

**Table 2:** Main and interaction effects for all psychophysiological responses and kinematic profiles.

Variable	Format Effect [95% CI]; F; *p*; $\eta_{p}^{2}$	Supplementation Effect [95% CI]; F; *p*; $\eta_{p}^{2}$	Format × Supplementation [95% CI]; F; *p*; $\eta_{p}^{2}$
PL (A.U.)	[−33.20, −29.79]; F = 1463.3; *p* < 0.001; $\eta_{p}^{2}$ = 0.985	[7.48, 13.24]; F = 55.3; *p* < 0.001; $\eta_{p}^{2}$ = 0.706	[5.74, 9.40]; F = 73.1; *p* < 0.001; $\eta_{p}^{2}$ = 0.761
TD (m)	[−172.77, −128.87]; F = 202.0; *p* < 0.001; $\eta_{p}^{2}$ = 0.898	[64.23, 142.73]; F = 29.7; *p* < 0.001; $\eta_{p}^{2}$ = 0.564	[2.08, 92.67]; F = 4.7; *p* = 0.041; $\eta_{p}^{2}$ = 0.169
AV (km·h^−1^)	[−2.08, −1.96]; F = 4202.9; *p* < 0.001; $\eta_{p}^{2}$ = 0.995	[0.34, 0.52]; F = 91.1; *p* < 0.001; $\eta_{p}^{2}$ = 0.798	[0.08, 0.33]; F = 10.8; *p* = 0.003; $\eta_{p}^{2}$ = 0.320
ACC (*n*)	[−16.61, −15.14]; F = 1983.8; *p* < 0.001; $\eta_{p}^{2}$ = 0.989	[2.61, 6.22]; F = 25.6; *p* < 0.001; $\eta_{p}^{2}$ = 0.526	[0.89, 2.28]; F = 22.4; *p* < 0.001; $\eta_{p}^{2}$ = 0.493
HR_mean_ (beats·min^−1^)	[−18, −17]; F = 4442.3; *p* < 0.001; $\eta_{p}^{2}$ = 0.995	[3, 7]; F = 30.3; *p* < 0.001; $\eta_{p}^{2}$ = 0.569	[0, 2]; F = 4.5; *p* = 0.044; $\eta_{p}^{2}$ = 0.164
HR_max_ (beats·min^−1^)	[−19, −17]; F = 1046.4; *p* < 0.001; $\eta_{p}^{2}$ = 0.978	[3, 6]; F = 53.4; *p* < 0.001; $\eta_{p}^{2}$ = 0.699	[1, 4]; F = 4.6; *p* = 0.044; $\eta_{p}^{2}$ = 0.166
%HR_max_ (%)	[−2.08, −1.60]; F = 257.1; *p* < 0.001; $\eta_{p}^{2}$ = 0.918	[0.90, 1.32]; F = 115.8; *p* < 0.001; $\eta_{p}^{2}$ = 0.834	[−1.32, −0.04]; F = 4.9; *p* = 0.037; $\eta_{p}^{2}$ = 0.176
Z0 (m)	[29.07, 40.41]; F = 160.7; *p* < 0.001; $\eta_{p}^{2}$ = 0.875	[0.24, 43.66]; F = 4.4; *p* = 0.048; $\eta_{p}^{2}$ = 0.160	[−41.20, −18.70]; F = 30.3; *p* < 0.001; $\eta_{p}^{2}$ = 0.569
Z1 (m)	[20.48, 29.78]; F = 125.0; *p* < 0.001; $\eta_{p}^{2}$ = 0.845	[15.70, 25.26]; F = 78.5; *p* < 0.001; $\eta_{p}^{2}$ = 0.773	[−62.42, −0.92]; F = 4.5; *p* = 0.044; $\eta_{p}^{2}$ = 0.165
Z2 (m)	[−53.93, −47.72]; F = 1147.5; *p* < 0.001; $\eta_{p}^{2}$ = 0.980	[2.58, 33.82]; F = 5.8; *p* = 0.024; $\eta_{p}^{2}$ = 0.202	[1.23, 15.29]; F = 5.9; *p* = 0.023; $\eta_{p}^{2}$ = 0.204
Z3 (m)	[−96.30, −79.45]; F = 465.8; *p* < 0.001; $\eta_{p}^{2}$ = 0.953	[9.84, 21.69]; F = 30.3; *p* < 0.001; $\eta_{p}^{2}$ = 0.569	[9.22, 27.03]; F = 17.7; *p* < 0.001; $\eta_{p}^{2}$ = 0.435
Z4 (m)	[−36.94, −28.30]; F = 244.0; *p* < 0.001; $\eta_{p}^{2}$ = 0.914	[10.62, 17.56]; F = 70.7; *p* < 0.001; $\eta_{p}^{2}$ = 0.755	[5.06, 11.95]; F = 26.0; *p* < 0.001; $\eta_{p}^{2}$ = 0.531
Z5 (m)	[−7.86, −4.95]; F = 83.1; *p* < 0.001; $\eta_{p}^{2}$ = 0.783	[4.32, 6.06]; F = 153.2; *p* < 0.001; $\eta_{p}^{2}$ = 0.869	[0.86, 3.62]; F = 11.2; *p* = 0.003; $\eta_{p}^{2}$ = 0.328
RPE (AU)	[1.40, 2.48]; F = 55.0; *p* < 0.001; $\eta_{p}^{2}$ = 0.705	[−1.95, −1.34]; F = 127.4; *p* < 0.001; $\eta_{p}^{2}$ = 0.847	[0.37, 1.55]; F = 11.3; *p* = 0.003; $\eta_{p}^{2}$ = 0.329
EES (score)	[−6.72, −3.58]; F = 45.9; *p* < 0.001; $\eta_{p}^{2}$ = 0.666	[6.73, 10.81]; F = 79.0; *p* < 0.001; $\eta_{p}^{2}$ = 0.775	[−2.66, −0.09]; F = 4.9; *p* = 0.037; $\eta_{p}^{2}$ = 0.175
VAS (score)	[1.27, 1.94]; F = 98.0; *p* < 0.001; $\eta_{p}^{2}$ = 0.810	[−2.04, −1.59]; F = 283.1; *p* < 0.001; $\eta_{p}^{2}$ = 0.925	[0.04, 1.21]; F = 4.9; *p* = 0.036; $\eta_{p}^{2}$ = 0.177

Note. CI = confidence interval; $\eta_{p}^{2}$ = partial eta squared; PL = player load; TD = total distance; ACC = accelerations; HR = heart rate; Z0 = 0–11 km·h^−1^; Z1 = 11–14 km·h^−1^; Z2 = 14–17 km·h^−1^; Z3 = 17–21 km·h^−1^; Z4 = 21–24 km·h^−1^; Z5 = >24 km·h^−1^ ; RPE = rating of perceived exertion; EES = exercise enjoyment scale; VAS = visual analogue scale; format effect = CON–INT; supplementation effect = CAF–PLA; format × supplementation = [(CAF-INT–PLA-INT) − (CAF-CON–PLA-CON)].

## Data Availability

The dataset supporting the findings of this study is available as a Supporting Information file.

## Supplementary Materials

The following supporting information can be downloaded at: https://www.mdpi.com/article/10.3390/nu18121962/s1.

## Abbreviations

The following abbreviations are used in this manuscript:

CAF	Caffeinated
INT	Intermittent
CON	Continuous
SSG	Small-sided game
PLA	Placebo
HR	Heart rate
RPE	Rating of perceived exertion
EES	Exercise enjoyment scale
MF	Mental fatigue
YYIRT-1	Yo-Yo Intermittent Recovery Test Level 1
VO_2max_	Maximal oxygen uptake
VAS	Visual analogue scale
PL	Player load
TD	Total distance
ACC	Accelerations
SD	Standard deviation
